# Longitudinal Associations Between Neighborhood Disorder, Diabetes, and Depressive Symptoms in Later Life

**DOI:** 10.1093/geroni/igaf122.2170

**Published:** 2025-12-31

**Authors:** Seungjong Cho, Jiao Yu

**Affiliations:** Texas Tech University, Lubbock, Texas, United States; Yale University, New Haven, Connecticut, United States

## Abstract

Neighborhood environments are increasingly recognized as determinants of mental health, yet the biological pathways linking neighborhood stressors to depressive symptomatology remain underexplored. Chronic exposure to neighborhood physical disorder, characterized by visible signs of neglect such as vandalism and poorly maintained infrastructure, may activate physiological stress responses that disrupt metabolic regulation, elevating the risk of diabetes and related complications. Moreover, diabetes-related metabolic dysregulation has been connected to the development of depressive symptoms, yet the underlying mechanisms warrant further investigation. To address this gap, we examined whether metabolic dysregulation, measured by hemoglobin A1c (HbA1c), mediates the association between perceived neighborhood physical disorder and depressive symptoms. Using longitudinal data from the Health and Retirement Study (HRS) Left-Behind Questionnaire, we analyzed three waves spanning 2006 to 2016 (N = 5,576). Perceived neighborhood physical disorder (2006/2008) was hypothesized to influence depressive symptoms (2014/2016) through HbA1c levels (2010/2012). Covariates included baseline depressive symptoms, age, gender, education, income, and marital status. A structural equation modeling path model (SRMR=.020) revealed a significant indirect path: greater neighborhood physical disorder was associated with higher HbA1c (Coef=.051, SE=.013, p<.001), which in turn predicted elevated depressive symptoms (Coef=.057, SE=.028, p=.046). These findings suggest that diabetes and related complications may serve as a physiological pathway through which neighborhood stressors impact mental health. Given the role of HbA1c in both metabolic and mental health, interventions targeting neighborhood disorders could have broader health benefits beyond social and psychological well-being. Future research should investigate additional biological pathways and explore interventions that address neighborhood stressors to promote healthier aging.

